# Objective and perceived food environment and household economic resources related to food insecurity in older adults living alone in rural areas

**DOI:** 10.1186/s12877-019-1231-y

**Published:** 2019-08-27

**Authors:** Jae Eun Shim, Ji-Yun Hwang, Kirang Kim

**Affiliations:** 1 Department of Food and Nutrition, Daejeon University, Daejeon, 34520 South Korea; 2 Department of Foodservice Management and Nutrition, Sangmyung University, Seoul, 03016 South Korea; 3 Department of Food Science and Nutrition, Dankook University, Cheonan, 31116 South Korea

**Keywords:** Food insecurity, Food environment, Economic factors, Older adults, rural area

## Abstract

**Background:**

Limited attention has been paid to an association between food environment and household economic resources related to food expenditure in food-insecure seniors. The aim of the study was to investigate the relationship between factors of economic resource, food environment, and food insecurity in single seniors residing in rural areas of South Korea.

**Methods:**

A cross-sectional study was conducted in 170 single senior households aged 65 years or over residing in rural areas. Face-to-face interviews were performed to collect data on demographic characteristics, household economic resources/expenditure, food environmental factors, and food insecurity.

**Results:**

Among economic resources, generally limited food expenditures due to housing fees and heating costs during the winter were positively related to food insecurity. Among food environmental factors, food accessibility at community level such as food stores located far from home and inconvenient bus routes was related to food insecurity. The most explainable economic and food environment factors related to food insecurity by stepwise logistic regression analysis were the percentage of total expenditure on housing fee (OR = 1.021, 95% CI: 1.008–1.034), foods purchasing at super supermarket (OR = 0.398, 95% CI: 0.166–0.951), having difficulties in food purchasing due to food stores being located far from home (OR = 14.487, 95% CI: 5.139–40.842) and inconvenient bus routes (OR = 0.083, 95% CI: 0.015–0.460).

**Conclusion:**

Inadequate community food environment as well as limited household food resources were an important risk factor for food insecurity in Korean single rural seniors. Findings of this study could help us better understand how characteristics of household food resources and community food environment can serve as barriers or facilitators of food security among single older adults residing in rural areas.

## Background

Food insecurity in the elderly living alone has recently attracted substantial research interest due to increasing number of older adults in developed countries. Food insecurity refers to a lack of available financial resources for food at household level. In 2012, the nationwide prevalence of food insecurity was 11.3% in total population of Korea and 13.3% in older adults [1]. Specifically, the nationwide prevalence of food insecurity in low-income household with elders aged ≥65 years was 56.2% [2].

Korea is one of the most rapidly aging developed countries due to a combination of extremely low birth rates and increasing life expectancy, with those aged 65 years or older accounting for 12.7% of the population in 2014 [3]. Furthermore, elderly households constitute 20% of all households [4]. The number of older people who live alone has rapidly increased (by 1320%) from 1985 to 2010 [5]. The proportion of older Koreans living in rural communities is 36% [4], although Korea is highly urbanized, with 82% of the population living in cities [6]. Compared to older adults living in cities, those living in rural areas are known to have significantly more health problems due to inadequate access to health care services and resources [7–9]. These results suggest it would be crucial to set priority of public health interventions for rural older adults living alone.

Several studies have reported that seniors living in rural areas have limited access to food stores that accommodate healthy food choice [10–12]. In this regard, older seniors with food insecurity are experiencing difficulties meeting their food needs. Thus, they are at high risk for malnutrition and other chronic diseases [11, 13, 14]. Features of single seniors living in rural areas such as physical immobility, lack of cooking skills, and lack of or limited food and/or non-food supports from any sources can exacerbate the association between food insecurity and chronic diseases. However, information on the risk of food insecurity in relation to household economic resources and food environment in Asian elderly population living alone in rural areas is very limited. To the best of our knowledge, only one study [15] has provided information about food purchase availability and accessibility for rural households including elderly people in South Korea, although South Korea is considered to have the most rapidly aging population in the world. However, this previous study did not target older adults living alone. It did not consider both economic resources and food environment to explain food insecurity in the study model either.

Recently, attention to measures of food environment is increasing due to increasing importance of food environment. Specifically, a variety of methodologies including objective and respondent-based perceived measures have been used to determine the degree of food access [16]. However, several studies have shown inconsistent results using different measures of food environment [17–21]. In addition, few studies have used both perceived and objective measures in their studies [18–20]. To better capture multiple dimensions of food environment, combining multiple environmental assessments needs to be considered. Therefore, the objective of this study was to investigate the relationship of economic resources and food environments with food insecurity for seniors living alone in rural areas of South Korea using both objective and perceived measures.

## Methods

### Design and participants

A cross-sectional study was conducted in rural areas of South Korea: Yangpyeong County in Gyeonggi Province and Hongcheon County in Gangwon Province. These two regions were selected for review by geographers, nutritionists, and public health professionals to demonstrate diverse characteristics of rural areas such as land and mountain areas. Yangpyeong County is 45 km east of Seoul, the capital of South Korea. It is a designated agricultural area with regulatory exemptions under the Environment-Friendly Agriculture Fosterage Act. Hongcheon County is 81 km northeast of Seoul. It is a mountainous region that flows from the Bakdu Mountain in the center of the Korean peninsula and forms a canyon. In this study, Yangpyeong County represents the land area while Hongcheon County represents the mountain area. Both regions have similar age distributions to other rural areas. Subjects were recruited by nurses of the National Home Healthcare Services (NHHS). The NHHS is a service that allows home-visiting nurses in public centers to visit vulnerable population with health problems and manage their health [22]. Priority registration for the NHHS applies to households with a monthly income below 50% of the median income [22].

Among subjects in the NHHS, home visiting nurses recruited older adults by the following inclusion criteria: 1) aged 65 years or more; 2) health conditions without cognitive impairment; 3) living at home at the time of the assessment; and 4) provided informed consent. All subjects received a telephone call or a face-to-fact contact. They were recruited by their home visiting nurses of public health centers in Yangpyeong and Hongcheon counties between November and December 2013. These two study areas had similar household economic characteristics, including food insecurity (40.0% in Yangpyeong County and 46.3% Hongcheon County, data not shown) and average monthly income for the last year. Upon agreement to participate in this study, all subjects received a phone call or a text message at least 3 times inviting them to attend a comprehensive fact-to-face interview. The base population was about 900 households with older adults receiving the NHHS (9 towns from Yangpyeong and 2 towns from Hongcheon). A total of 170 rural seniors without disability living alone finally completed the survey. Therefore, our study samples covered 18.9% of the study population. A well-trained survey team was composed of professionally experienced interviewers in this study. To ensure uniformity, two head interviewers underwent 1 day of training by a principal investigator of the study with a standardized study protocol. All interviewers were required to attend 2 days of training by head interviewers before the survey was initiated. Informed written consent for participation was obtained from each individual. This study was approved by the Institutional Review Board of Sangmyung University, Seoul, Korea (approval number: BE2013–8).

### Measurements

Detailed data on demographic characteristics, household economic resources/expenditure, food environmental factors, and food insecurity were collected for each study participant. Demographic variables were age, sex, education, employment, and beneficiary of national basic livelihood. The national basic livelihood is a service of monetary payment for daily necessities such as food, clothing, and fuel for low-income families. It is defined as the amount of the reported household income deducted from the minimum level of livelihood wage (30% of the median income). Objective and perceived measures of economic resources and food environments are described as below.

### Household economic factors

Objective household economic indices of monetary incomes and expenditures per month for food and non-food (housing, heating and medical expenses) were collected in Korean Won (the exchange rate of currency: 1125 Korean won = 1 US dollar). The distribution of household consumption expenditure (percentage of total) from each monetary term was calculated. Perceived indicators of household economic experience were measured by asking whether they reduced food expenditure due to the burden of non-food expenditures such as housing fee and heating costs during a winter season.

### Food environment factors

Food environment conceptualizations in this study were developed based on the concept model of Glanz and colleagues [23]. This model organizes food environment features into community food environment (the distribution of food sources within a community such as number and accessibility of food outlets) based on an ecological model of health behaviors. The organizational food environment (the multiple settings where people eat or procure food such as home, school, work, and others) and consumer food environment (available healthy food options and the cost and quality of foods in local food outlets) are contributors to healthy eating patterns. These food environments could be moderated or mediated by social environment [24]. Especially, for older adults living in rural areas, limited food availability and accessibility due to financial limitations, physical limitations, and inability to drive should be addressed by social supports [25–27].

Based on the literature, this model includes two aspects of availability and accessibility of food at household and community levels. The assessment of food availability was focused on the supply source of available food and the presence of social supports to help ensure food supplies at household level and the presence of various foods at community level. Household-level food accessibility included the presence of social supports helping access to foods while community-level food accessibility included geographic food access such as location of food store and ease of getting to that location. Because there was no best measurement concerning the definition of food environment, we measured food environment using questionnaires including both objective observational and perceived dimension as proposed in several previous studies [18, 28, 29].

The questionnaire-based measurement of food environment was as follows. Household food availability was obtained objectively by food acquisition methods such as food purchase, farming or home gardening, tangible private food, and beneficiaries of public food assistance programs. Household food accessibility was measured by asking subjects whether there were intangible supports related to food purchase from a family and/or neighbors existing for subjects enrolled for this study.

Community food availability and accessibility were examined using objective or perceptional-based measurements. Community food availability was measured by asking them whether subjects purchased foods mostly and by asking them in detail their perceptions on whether the nearest food store accommodated various purchasable food items to meet their needs. Community food accessibility was measured objectively by transportation and distance to get to the nearest food stores. Perceived community food accessibility was measured by determining whether subjects experienced any difficulty in food purchasing due to long distance to food stores from home, a bus stop being located in a remote location from home base, or inconvenient bus routes.

### Household food insecurity

Household food insecurity was measured using the validated Korean Household Food Security Survey Module (K-HFSS) from the Korea National Health Examination and Nutrition Survey [30, 31]. The K-HFSS was based on the 18-item US Household Food Security Survey Module (HFSSM). This 18-item questionnaire consisted of 3 household-referenced questions, 7 adult-referenced questions, and 8 child-referenced questions. In the present study, the adult food security survey module consisting of 10-item questionnaire (3 household-referenced questions and 7 adult-referenced questions) was completed by each household. A score of 1 was allocated to affirmative responses to each item and a score 0 was assigned otherwise. Subjects with scores of 3–10 were classified as having food insecurity.

### Statistical analysis

Data are presented as percentage and number for categorical variables and mean ± SD for continuous variables. Results were compared between food-secure and food-insecure households using Chi-square test for categorical variables and t-test for continuous variables. Multivariable-adjusted logistic regression was conducted to determine odds ratios (OR) and 95% confidence intervals (CI) for a risk of food insecurity. Stepwise logistic regression was used to identify the most explainable economic and food environmental factors, including all the variables listed as demographic, economic, and food environment characteristics. All analyses were performed using IBM SPSS Statistics 23 (IBM Company, Armonk, NY, USA). Statistical significance was defined at *p* < 0.05.

## Results

### Demographic characteristics of single senior households

Demographic characteristics are presented in Table 1. The prevalence of food insecurity was 34.7% (*n* = 59) in the study sample. The mean age of subjects was 77.6 years. Most participants were women (84.7%) and unemployed (91.8%). Majority (90.6%) of them had less than a middle school education. About 44% of participants were beneficiaries of the national basic livelihood. There was no significant difference in age, sex, education, employment, or beneficiaries of national basic livelihood between food-secure and food-insecure households.

**Table 1:** Demographic characteristics of single person households according to food security status

Variables	Total	Food secure household	Food insecure household	t or chi-square
	*n* = 170	*n* = 111	*n* = 59	
Age (years)	77.6±6.5	77.5±6.9	77.6±5.8	0.00
women	84.7(144)	84.7(94)	84.8(50)	0.00
≤6 years of primary education	90.6(154)	91.9(102)	88.1(52)	0.64
Having job	8.2(14)	10.8(12)	3.4(2)	2.81
Beneficiaries of national basic livelihood	44.1(75)	41.4(46)	49.2(29)	3.45

Values are mean ± SD for continuous variables or % (n) for categorical variables

### Economic characteristics related to food insecurity

Economic characteristics of single senior households according to food insecurity are shown in Table 2. For objective economic indices, monthly housing fee in real terms ($66.4 vs. $31.3, *P* < 0.01) and percentage compared to total expenditure (29.4% vs. 14.1%, *P* < 0.01) was higher for food-insecure households than that for food-secure households. In contrast, monthly medical expenditure in monetary amount ($30.9 vs. $14.7, *P* < 0.05) and percentage compared to total expenditure (18.3% vs. 9.5%, *P* < 0.05) was higher for food-secure households than that for food-insecure households. For subjective economic indices, the experience of reducing food expenditure resulting from burden of housing fee (*p* < 0.01) and heating costs during winter (*p* < 0.01) were higher for households with food insecurity than those for households without food insecurity.

**Table 2:** Economic characteristics of single older adults according to food security status

Variables	Total	Food secure household	Food insecure household	t or chi-square
Objective economic indices				
Average monthly income for last 1 year, $ ^a^	275.5±149.9	280.9±156.6	265.3±137.2	0.42
Earnings	44.7±100.5	53.1±111.9	28.9±72.8	2.24
Subsidies	189.6±139.9	182.6±141.9	202.9±136.4	0.81
Allowances from family	41.2±88.1	45.2±94.1	33.4±75.6	0.69
Average monthly expenditure for last 1 year, $	157.8±101.2	153.7±100.8	165.6±102.2	0.54
Food expenses	36.0±41.3	37.5±44.1	33.2±35.5	0.41
Housing fee	43.5±71.7	31.3±64.4	66.4±79.3	9.75**
Heating costs	53.0±36.3	54.0±36.8	51.2±35.6	0.22
Medical expenses	25.3±42.9	30.9±48.1	14.7±28.6	5.61*
% Proportions of expenditure components, all year				
Food expenditure	22.9±19.5	24.8±20.6	19.3±16.7	3.05
Housing fee	19.4±29.7	14.1±26.1	29.4±33.4	10.8**
Heating costs	41.9±26.5	42.9±25.4	40.1±28.8	0.42
Medical expenditure	15.3±21.5	18.3±22.4	9.5±18.4	6.63*
Perceived economic indices				
Reduced food expenses due to burden of housing fee	21.8(37)	15.3(17)	33.9(20)	7.82**
Reduced food expenses due to burden of heating costs	51.8(88)	44.1(49)	66.1(39)	7.44**

Values are mean ± SD for continuous variables or % (n) for categorical variables

* significantly different between food secure and insecure households (*p < 0.05, **p < 0.01)

^a^ The exchange rate of currency was 1125 Korean won per 1 US dollar

### Food environmental characteristics related to food insecurity

Table 3 shows food environmental characteristics at household and community levels according to food insecurity. Among household food availability and accessibility factors, household with public food assistance program had higher proportion in household with food insecurity than that in household with food security (66.1% vs. 45.1%, *p* < 0.01). The proportion of household that acquired foods by using farming or home gardening resources (24.3% vs. 11.9%, *p* = 0.0532) and the proportion of household with intangible support for food purchasing from family (12.6% vs. 1.7%, *p* = 0.0502) tended to be greater in households with food security than those in households with food insecurity. Among relevant community food accessibility factors by perceived measurements, the proportion of households with difficulties in food purchasing due to food stores being located far from home was greater in households with food insecurity (44.1% vs. 16.2%, *p* < 0.001) whereas the proportion of households with difficulties in food purchasing due to inconvenient bus routes was greater in households without food insecurity (16.2% vs. 3.4%, *p* < 0.05).

**Table 3:** Food environmental characteristics of single older adults according to food security status

Variables	Total	Food secure household	Food insecure household	t or chi-square
Household food availability				
Purchasing food	82.4(140)	82.0(91)	83.1(49)	0.03
Farming or home gardening	20.0(34)	24.3(27)	11.9(7)	3.74
Private food assistance	18.8(32)	19.8(22)	17.0(10)	0.21
Public food assistance program	52.4(89)	45.1(50)	66.1(39)	6.85**
Household food accessibility				
Having intangible support for food purchasing from family	8.8(15)	12.6(14)	1.7(1)	5.98
Having intangible support for food purchasing from neighbors	6.5(11)	4.5(5)	10.2(6)	2.04
Community food availability, objective indices				
Places to purchase food				
Traditional market	35.3(60)	34.2(38)	37.3(22)	0.16
Super market	13.5(23)	11.7(13)	17.0(10)	0.90
Super supermarket	45.3(77)	48.7(54)	39.0(23)	2.02
Community food availability, perception^a^				
No various foods in the nearest food store	8.2(14)	5.4(6)	13.6(8)	3.40
Community food accessibility, objective indices				
Transportation to the nearest food stores^b^				0.59
By walk	43.5(74)	41.4(46)	47.5(28)	
By driving	38.8(66)	40.5(45)	35.6(21)	
Distance to the nearest food stores (min)				
By walk^c^	17.1±12.3	15.9±13.8	19.1±9.0	1.21
By driving^c^	20.7±9.1	21.7±8.5	18.6±10.1	1.67
Community food accessibility, perception^a^				
Having difficulties in food purchasing due to food stores far from home	25.9(44)	16.2(18)	44.1(26)	16.46***
Having difficulties in food purchasing due to bus stop far from home	15.9(27)	18.9(21)	10.2(6)	2.44
Having difficulties in food purchasing due to inconvenience bus route	11.8(20)	16.2(18)	3.4(2)	6.47*

Values are mean ± SD for continuous variables or % (n) for categorical variables

* significantly different between food secure and insecure household (*p < 0.05, **p < 0.01, ***p < 0.001)

^a^ Responses were “yes”, “no”, or “not applicable (non-purchasing of foods)”

^b^ Responses were “by walk”, “by driving”, or “not applicable”

^c^ Responses among the participants who used the transportation to the nearest food store

### Relationship of food insecurity with economic resources and food environment

The results of stepwise logistic regression to select the most explainable economic and food environmental factors related to food insecurity are shown in Table 4. Among economic factors, the percentage of total expenditure on housing fee was positively related to food insecurity (OR = 1.021, 95% CI: 1.008–1.034). Among food environmental factors, having difficulties in food purchasing due to food stores being located far from home (OR = 14.487, 95% CI: 5.139–40.842) and non-purchasing of foods regardless of such difficulties (OR = 5.946, 95% CI: 1.659–21.311) were positively related to food insecurity while foods purchasing at super supermarket (OR = 0.398, 95% CI: 0.166–0.951) and having difficulties in food purchasing due to inconvenient bus routes (OR = 0.083, 95% CI: 0.015–0.460) were negatively related to food insecurity.

**Table 4:** Risk factors for food insecurity in single older adults

Risk factors	Odds ratio	95% Confidence interval	
Economic indices			
Housing fee (%expenditure)	1.021	1.008	1.034
Food environmental indices			
Food purchasing at super supermarket^a^	0.398	0.166	0.951
Having difficulties in food purchasing due to food stores far from home^a^	14.487	5.139	40.842
Non-purchasing of foods^b^	5.946	1.659	21.311
Having difficulties in food purchasing due to inconvenience bus route^a^	0.083	0.015	0.460

Odds ratios and 95% confidence limits of risk factors for households’ food insecurity

Multivariable-adjusted logistic regression analysis was used and independent variables included all the variables listed as demographic, economic (%proportion for expenditure), and food environment characteristics. A stepwise approach was applied to select the most explainable risk factors in the model (α = 0.15)

^a^ Reference group was subjects who responded “no” to the question

^b^ Reference group was subjects who purchased foods without difficulties in food purchasing due to food stores far from home

## Discussion

Although food environments and household economic resources related to food expenditures have been concerned for food-insecure seniors, limited attention has been paid to these factors in an Asian cultural context. This study found that factors associated with food insecurity among older adults living alone in rural areas of South Korea were high housing expenditure, non-purchasing of foods at super supermarket, and having difficulties in food purchasing because of food stores located far from home or inconvenient bus routes.

In this study, single older adults with difficulty in food purchasing due to food stores located far from home were more likely to be food insecure than those without such situation. However, unexpectedly, those with difficulty in food purchasing due to inconvenient bus routes were less likely to be food insecure than those without such difficulty. In general, physical food access is a major problem for people with mobility disabilities such as older adults or people with low incomes without ownership of a vehicle [26, 32, 33]. In order to understand such unexpected result, we further compared other factors related to food insecurity between older adults with difficulty in food purchasing due to inconvenient bus routes and those without such difficulty. As a result, older adults with the difficulties in food purchasing were less likely to have the experience of reducing food expenditure resulting from burden of housing fee (0% vs. 27.5%, *p* = 0.0104) or heating costs (30% vs. 60%, *p* = 0.0038) and were more likely to have intangible support for food purchasing from family (25% vs. 8.33%, *p* = 0.0417) or use cars rather than a walk to buy foods (90% vs. 40%, *p <* 0.0001). These characteristics of older adults with the experience of difficulty in food purchasing due to inconvenient bus routes might affect being food-secure. However, the reverse-causal association should also be considered because this was a cross-sectional study. Food secure older adults might be more likely to perceive difficulty in food purchasing due to inconvenient bus routes.

Several studies have shown that older adults in rural areas are affected by poor access to food stores and healthy food items [27, 33–35]. These individuals might have difficulties to maintain healthy food intakes due to limited access to healthful food stores. Indeed, it has been found that older adults residing in rural areas consume inadequate amounts of fruits, vegetables, dairy products, and proteins [17, 20, 27]. A recent study has found that increased number and density of supermarkets in a neighborhood are associated with more consumption of healthy foods and lower body mass index or waist circumference [36]. Our study also found similar results that buying foods at super supermarkets where diverse, cheap and fresh foods are available was related to food security. Given that greater distance to food stores which could provide cheap and high quality foods was found to be a barrier for food security in Korean older adults residing in rural areas, future interventions need to consider strategies to address differential access to foods due to food store distant from home.

Especially, nutritional status in older adults has greater challenges due to their limited economic resources. According to a survey on the current livelihood status and the need for welfare in the Korean elderly, food expenditure accounted for the highest household expenditure in older adults [37]. In the present study, the high housing expenditure was considered to be one of the important barriers for determining food security. Because the changes in household resources allocation would result in worse outcomes, the study on understanding of how resources are allocated in the food-insecure elderly households is necessary. Housing assistance programs need to be considered for older adults living alone and struggling to pay their housing bills to reduce food insecurity.

Our study examined food environment using both objective and perceived (subjective) measures. Significant relationship of food insecurity with food environment was shown in perceived measures of community food accessibility: perceived difficulty in food purchasing due to the food stores far from an individual’s home and the inconvenient bus route. The importance of perceived measures of food environment has been reported in a previous study. The relationship of availability or accessibility of foods with food purchasing and intakes has been found to be more significant in perceived measures than that in objective measures, especially among adults with lower incomes [18]. However, another study on low income women has reported that objective food store environment measures, but not subjective measures, are associated with fruit and vegetable intakes [19]. Several studies have shown that both subjective and objective food environment measures are related to intakes of fruits and vegetables [17, 20]. A previous study has found that there is a low agreement between objective and perceived community nutrition environment measures due to different socioeconomic status [21]. This could imply that persons with low income including subjects enrolled for the present study might shop in stores outside of their local proximity to buy cheaper foods. This might be the reason why subjective measures of food environment could better explain the food environment than objective measures. Therefore, obtaining both measures (subjective and objective) are needed in future studies to well understand the food environment of vulnerable populations.

Interpretation of this study should also consider several study limitations. Since this study had a cross-sectional nature, a causal association between factors and food insecurity could not be determined. This study was localized to marginal rural areas in Korea. They could not be representative of all rural areas. Thus, it is difficult to generalize these findings to all older adults in Korea. Further studies in urban settings using more representative samples are needed to investigate whether there are different aspects of residence areas. Moreover, the relatively small sample size limits our ability to detect practically meaningful results, although results of our study showed statistical significance. Further studies with adequate sample size are needed to confirm our results. Despite several limitations, the key strength of this study is that this is the first study to examine simultaneously both food environmental factors and household economic resources in association with food insecurity among rural seniors living alone, especially in an Asian cultural context. In addition, we examined the relationship of food insecurity with food environment and economic resources using both objective and perceived measures.

## Conclusion

In conclusion, Korean single rural seniors with inadequate household food resources and community resources such as limited food expenditures due to high housing expenditure, limited access to super supermarkets, and living in an area that has a long distance from the nearest food store were at risk for food insecurity. Findings from our study could help us better understand how characteristics of household economic resources and community food environment could serve as barriers or facilitators for food security among older adults residing in rural areas. To improve nutrition-related health problems of this growing elderly population, interventions should include improvement of food environment as well as assistance programs to support need for basic livelihood such as housing assistance via social support system.

## Data Availability

Raw data collected in the study are only available to participating researchers due to risk to confidentiality of participants and data protection laws. An anonymized dataset (direct and potentially indirect person identifiers are removed) is available from the corresponding author upon reasonable request.

## Abbreviations

CI: Confidence interval
NHHS: National Home Healthcare Services
OR: Odds ratio
SD: Standard deviation
